# Investigating gray matter volumes in idiopathic epileptic Border Collies and Greater Swiss Mountain Dogs using voxel-based morphometry

**DOI:** 10.3389/fvets.2026.1872171

**Published:** 2026-07-17

**Authors:** Imaine Glahn, Henning Richter, Katrin Beckmann

**Affiliations:** 1 Diagnostic Imaging Research Unit, Clinic for Diagnostic Imaging, Vetsuisse Faculty, University of Zurich, Zurich, Switzerland; 2 Section of Neurology, Department of Small Animals, Vetsuisse Faculty, University of Zurich, Zurich, Switzerland

**Keywords:** dogs, gray matter, idiopathic epilepsy, MRI, voxel-based morphometry

## Abstract

**Objective:**

To investigate regional gray matter volume differences in Border Collies and Greater Swiss Mountain Dogs with idiopathic epilepsy compared with healthy controls using voxel-based morphometry (VBM).

**Methods:**

This retrospective single-center study analyzed 3D T1-weighted MRI datasets from 46 dogs, comparing dogs with idiopathic epilepsy with breed-matched healthy controls. Regional gray matter volume was assessed using an automated FSL-based voxel-based morphometry pipeline with semi-manual brain extraction and 1.5 mm smoothing kernel. To account for inter-breed anatomical variation, separate study-specific templates were created for each breed. Volumetric comparisons of gray matter, white matter, total brain and gray matter ratios were performed for both breeds and analyzed using the Mann–Whitney U test.

**Results:**

A total of 46 dogs were included, of those, 18 Border Collies and 13 Greater Swiss Mountain Dogs were diagnosed with idiopathic epilepsy, while 5 Border Collies and 10 Greater Swiss Mountain Dogs served as healthy controls. Volumetric comparisons highlighted pronounced size differences between breeds. Within each breed, no significant differences in gray and white matter volumes or ratios were found between healthy controls and epileptic dogs. A statistically significant difference in the gray matter ratio was detected when both breeds were pooled (58% vs. 57%, *p* = 0.039). VBM did not reveal any significant differences in gray matter volume between groups. However, exploratory analysis in Border Collies suggested increased gray matter volume in limbic regions (amygdala, retrosplenial fissure) and temporal areas, whereas Greater Swiss Mountain Dogs showed increases in the cerebellum. Both breeds showed focal gray matter volume reductions in the right anterior ectolateral gyrus.

**Conclusion:**

VBM analysis did not identify statistically significant differences in gray matter volume between dogs with idiopathic epilepsy and healthy controls. However, exploratory analyses revealed breed-specific patterns of both focal gray matter decreases and regional increases. This mixed pattern supports the concept of canine idiopathic epilepsy as a distributed network disease.

## 1 Introduction

Canine idiopathic epilepsy is characterized by recurrent epileptic seizures without identifiable structural brain abnormalities and is one of the most common chronic neurological diseases in dogs, with an estimated prevalence of 0.6–0.75% (1, 2). Several breeds are believed to have a genetic predisposition, including Border Collies (3) and Greater Swiss Mountain Dogs (4, 5).

The International Veterinary Epilepsy Task Force (IVETF) has defined criteria for the diagnosis of idiopathic epilepsy in a three-tier system, with increasing levels of diagnostic confidence for idiopathic epilepsy (6, 7). The diagnosis of idiopathic epilepsy can only be made if other diseases that trigger epileptic seizures, such as structural epilepsy (SE) or reactive seizures, have been ruled out. Therefore, magnetic resonance imaging (MRI) examination is routinely performed to exclude structural brain abnormalities including neoplasms, infections or vascular lesions (7). In addition to ruling out structural abnormalities, more advanced MRI techniques may serve as a tool for neuroscientific research and enable the detection of subtle brain parenchyma changes.

Advanced MRI based morphometric and volumetric neuroimaging studies have been used in in canine idiopathic epilepsy. These include manual volumetry (8), semi-automated volumetry (9), Cavalieri’s principle (10) and histogram analysis (11) and enable the detection of subtle changes and comparisons between study groups. However, many of these methods are time-consuming or limited to predefined regions of interest.

In contrast, Voxel-based morphometry (VBM) is a fully automated, computational, quantitative MRI analysis technique that allows for the detection of subtle volumetric differences in brain structures (12). VBM compares each voxel across groups of subjects after spatial normalization and segmentation into gray matter white matter, and cerebrospinal fluid compartments (12–14). It has been widely used in human medicine to detect localized changes in gray matter volume associated with neurological diseases, including temporal lobe epilepsy (12, 15) and idiopathic generalized epilepsy in humans (16).

In veterinary medicine, the application of VBM has been limited, partly due to the anatomical variability across dog breeds (17, 18). As a result, previous veterinary VBM research has primarily focused on single breed studies to minimize anatomical variation. A recent study of beagles with idiopathic epilepsy revealed that VBM analysis can detect significant gray matter reductions in regions such as the olfactory bulb, cingulate gyrus, and hippocampus (19), indicating that subtle structural brain changes may indeed exist despite normal conventional MRI findings (19, 20). In addition to its application in the field of canine epilepsy, VBM has been employed in the research of feline epilepsy (21), ventriculomegaly (22) or Chiari-like malformation (23), as well as in understanding normal and pathological aging (24, 25).

In light of the high prevalence of idiopathic epilepsy in Border Collies and Greater Swiss Mountain Dogs (3–5), the objective of this study was to assess gray matter volume using VBM in these breeds. We hypothesized that dogs with idiopathic epilepsy would exhibit focal differences in gray matter volume when compared to breed-matched healthy controls.

## 2 Materials and methods

### 2.1 Animals

This retrospective single-center study analyzed MRI data collected at the Vetsuisse Faculty of the University of Zurich between 2017 and 2022, following approval by the cantonal authorities according to Swiss law under animal license no. ZH272/16 and ZH046/20.

Border Collies and Greater Swiss Mountain Dogs were included in the study, if diagnosed with idiopathic epilepsy based on IVETF TIER II confidence level and excluded when younger than 1 year and older than 10 years to eliminate the influence of incomplete maturation and aging (26, 27).

According to previous studies (28–30), healthy controls (first degree relatives) were included if they had no history of seizures and no detected seizures within a follow-up time of 2.5 years after initial examination. Animals classified as healthy underwent clinical and neurological examination, a comprehensive blood chemistry, hematological laboratory examination and an MRI scan, which revealed no abnormalities.

Functional MRI and magnetic resonance spectroscopy data from most of these Border Collies and Greater Swiss Mountain Dogs with idiopathic epilepsy and healthy controls have also been analyzed in previous studies (29–31). Exclusion criteria were insufficient quality of image data.

### 2.2 Anesthesia during image acquisition

Image acquisition was performed under general anesthesia following a standardized anesthetic protocol as previously described in detail by Beckmann et al. (32). Briefly, premedication consisted of intramuscular butorphanol (0.1–0.2 mg/kg), followed by induction with propofol to effect and maintenance with sevoflurane in oxygen and medical air. Monitoring and supportive care, including normocapnia maintenance and blood pressure management, were conducted identically to the aforementioned study.

### 2.3 Magnetic resonance imaging

The epilepsy MRI protocol was performed with the dogs in a supine position using a 3-Tesla MRI scanner (Philips Ingenia, Philips AG, Zurich, Switzerland) and a 16-channel receive-transmit head coil (dStream HeadSpine coil solution, Philips AG, Zurich, Switzerland). The images analyzed in this study were acquired using a non-contrast 3D T1-weighted sequence (T1W; TR 8.7–10.2 ms; TE 3.8–4.7 ms; FOV 130 mm; slice thickness 0.6–0.8 mm; flip angle 8°; acquisition time 374.66–562.96 s), for anatomical evaluation. To accommodate the varying head sizes among dog breeds, the field of view was adapted individually for Border Collies and Greater Swiss Mountain Dogs. This resulted in a reconstructed acquisition matrix ranging from 288 × 288 to 336 × 336.

### 2.4 Preprocessing

**Figure 1:**
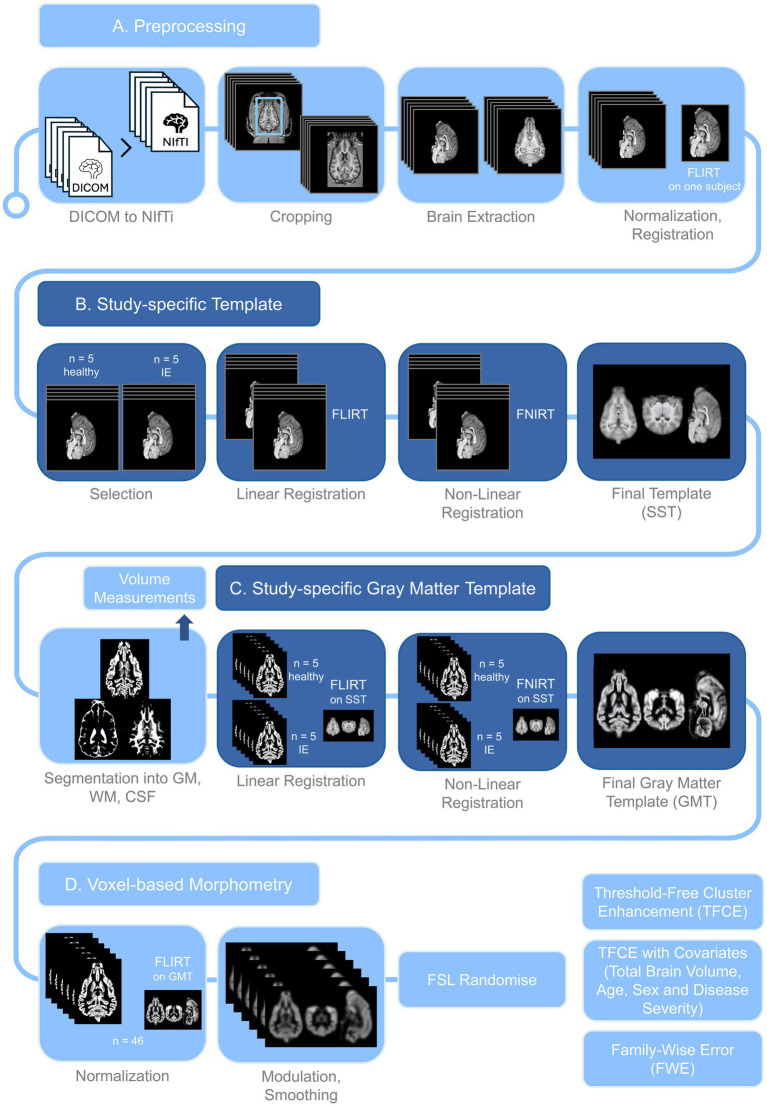
Voxel-based morphometry workflow: **(A)** Preprocessing performed separately for both Border Collies and Greater Swiss Mountain Dogs including cropping, brain extraction and normalization. **(B)** Selection of 10 subjects per breed to create study-specific templates. **(C)** Tissue segmentation and creation of study-specific gray matter templates. **(D)** VBM pipeline: normalization to gray matter template, modulation and smoothing, followed by statistical analysis using FSL Randomise with TFCE with/without covariates and FWE-correction. Volume measurements were derived by integrating voxel-wise probability values from segmented images. Light blue indicates steps for all subjects within their respective breeds; dark blue indicates steps for selected subjects for template creation. IE, Idiopathic Epilepsy; GM, Gray Matter; WM, White Matter; CSF, Cerebrospinal Fluid; SST, Study-specific Template; GMT, Gray Matter Template; TFCE, Threshold-free Cluster Enhancement; FWE, Family Wise Error.

Images from all dogs underwent a preprocessing pipeline that included conversion of DICOM images to NIFTI format using the MRIcron software package (version 1.0.20201102),^1^ brain extraction, initial normalization and registration. After initial normalization and registration to a single subject of the respective breed, the images of all dogs from both breeds were segmented into gray matter, white matter and cerebrospinal fluid using FSL FAST (33). Selected subjects of each breed were used to create study-specific, tissue-type templates, as using custom templates can yield more biologically plausible disease patterns than generic MNI templates (34). This pipeline was conducted separately for Border Collies and Greater Swiss Mountain Dogs, ensuring that all subsequent VBM analyses remained breed-specific (Figure 1).

#### 2.4.1 Brain extraction

To prepare for brain extraction, images were cropped to the relevant anatomical regions using the Crop Volume module in 3D Slicer (version 5.6.2)^2^ (35). A manual region of interest box was defined to exclude peripheral non-brain tissue, thereby improving the performance of subsequent preprocessing steps. These cropped images were then processed using FSL (FMRIB Software Library version 6.0.7.16, Oxford, UK) (36–38). The initial brain extraction masks, generated using FSL BET (39), showed the challenges commonly associated with brain extraction in non-human images. Specifically, regions of the canine brain, including the rhinencephalon, were incorrectly removed because these areas differ morphologically from the human brain.

To address this issue, the resulting brain extraction masks were manually refined in ITK-SNAP (version 4.2.2)^3^ (40) using the Active Contour Segmentation Mode. The segmentation process was initiated using the FSL BET segmentation output. The pre-segmentation mode was configured to “Threshold” using the threshold mode “upper and lower threshold.” The lower threshold was set to a value between 259 and 4,779, and the upper threshold was set to a value between 1,683 and 15,045. These Values were selected to optimize separation of brain tissue from surrounding structures. Contour initialization was conducted by placing one to seven bubbles in the image. The contour evolution of the segmentation was performed with an iteration value ranging from 41 to 326. Subsequently, the masks were visually inspected and, when necessary, manually corrected using the paintbrush inspector tool.

#### 2.4.2 Study-specific template

Study-specific template creation was performed in separate pipelines for Border Collies and Greater Swiss Mountain Dogs to provide an anatomically tailored reference space for each breed. All brain-extracted images were intensity normalized and registered using linear affine registration to a single subject within each breed to establish an initial common space. The subjects were selected based on their representative anatomy and image quality, as well as its dimensional size, with each group comprising an equal number of healthy controls and dogs with idiopathic epilepsy (*n* = 5). This balanced template, which included equal numbers of subjects from each group, helped prevent registration bias toward either group.

The template creation comprised three iterations of registration and template construction. A first template was created by merging and averaging the selected 10 subject images using FSL. Then a linear registration using FLIRT (41, 42) was conducted with the first template as a reference. Subsequently, the initial data were non-linearly registered using FNIRT (43) to the first template, utilizing the .mat files from the FLIRT step. Resulting images were merged, averaged, flipped and re-averaged to obtain the final study-specific template.

#### 2.4.3 Tissue-type template

The 10 subjects selected for the study-specific template were used to create a tissue-type template. Template creation followed the same steps consisting of one FLIRT and one FNIRT iteration (44). The previously obtained study-specific template was used as the reference for FLIRT registration to ensure accurate alignment within each cohort. Lastly the non-linearly registered images were merged, averaged, flipped and re-averaged to obtain a symmetrical final tissue-type template. This tissue-type template was methodologically required within the FSL VBM framework because it served as a contrast-matched target for non-linear registration and defined the final statistical mask within gray matter tissue.

### 2.5 Voxel-based morphometry

Thereafter, gray matter partial volume estimation maps of all dogs were registered to the breed-specific tissue template. To compensate for the structural displacement and geometric scaling inherent in non-linear registration, each voxel intensity in the individual gray matter images was multiplied by the local Jacobian determinant derived from the non-linear deformation fields. The Jacobian determinant mathematically quantifies the local volume change, reflecting regional expansion or contraction that occurs during spatial normalization. This modulation step is essential for ensuring that the subsequent voxel-based statistical analysis strictly evaluates absolute regional tissue volume rather than local tissue density. Subsequently, the data were smoothed using an isotropic Gaussian kernel with a sigma of 1.5 mm, corresponding to approximately three times the average voxel size (0.5 mm).

The resulting data were merged into two 4D files, one for each breed, each comprising the gray matter-PVE maps of both healthy controls and dogs with idiopathic epilepsy.

Statistical analysis was performed using FSL Randomize, a non-parametric permutation-based tool, to identify significant differences in gray matter volume between the groups. The analysis employed Threshold-Free Cluster Enhancement (TFCE) to detect significant clusters and Family-Wise Error (FWE) correction to account for multiple comparisons (*p* < 0.05). To account for individual differences in head size, analyses were conducted both with and without total brain volume, defined as the sum of gray matter and white matter, as a covariate. Absolute volumes for gray matter and white matter were derived by summing the partial volume estimates per voxel multiplied by voxel volume using FSL Stats; total brain volume was calculated by integrating the voxel-wise probability values from the FAST-generated gray matter-PVE maps, calculated as non-zero mean intensity multiplied by volume using FSL Stats, ensuring that partial volume effects were correctly accounted for. The General Linear Model was defined using four T-contrasts: healthy group mean (1 0); idiopathic epilepsy (IE) group mean (0 1); healthy > IE (1 −1), identifying areas of decreased gray matter in the IE group and IE > healthy (−1 1), identifying areas of increased gray matter volume in the IE group. Furthermore, additional analyses were carried out using age as a mean-centered continuous covariate, sex as a binary factor and disease severity as a categorical covariate. Covariate analyses were conducted separately to maintain statistical power. Disease severity was evaluated by categorizing the Border Collies into three groups: (1) healthy controls, (2) newly diagnosed or mildly affected dogs (defined as drug-naïve dogs with no reported status epilepticus and/or cluster seizures) representing a milder disease phenotype, and (3) severely affected dogs (defined by the use of at least two antiseizure medications, indicating a potential drug-resistant phenotype, and a history of status epilepticus and/or cluster seizures). Three dogs were excluded from this specific sub-analysis as they did not strictly fit into any of the predefined severity groups. Covariate analysis for disease severity was not performed in Greater Swiss Mountain Dogs, as the majority of these dogs were newly diagnosed and drug-naïve.

### 2.6 Statistical analysis

Descriptive statistics and bivariate comparisons were performed to characterize differences between dogs with idiopathic epilepsy and the healthy controls. For group comparisons, the Mann–Whitney U test was used. Categorical and binary variables were analyzed using Fisher’s exact test. Continuous data are presented as means with standard deviations (SD) or medians with interquartile ranges (IQR), depending on their distribution. Categorical variables are reported as frequencies and percentages.

Statistical significance was determined based on the aforementioned bivariate tests, with *p*-values < 0.05 considered statistically significant. All analyses were performed using Stata (Stata/MP18, StataCorp, TX, USA).

## 3 Results

### 3.1 Study population

A total of 48 dogs were included initially. Two Greater Swiss Mountain Dogs were excluded from the analysis due to inadequate raw data quality. Of these 46 dogs, 31 dogs (67%) had idiopathic epilepsy based on IVETF TIER II confidence level (7), while 15 dogs (33%) were unaffected. The epilepsy group included 18 Border Collies and 13 Greater Swiss Mountain Dogs. Except for one Border Collie, all of the healthy control dogs (*n* = 15) had either a littermate or a direct offspring affected by epilepsy. The study cohort consisted of 23 Border Collies and 23 Greater Swiss Mountain Dogs (Table 1). The mean age was 70 months (SD, 43) for the healthy Border Collies and 49 months (SD, 25) for epileptic Border Collies (overall: 54 months; SD, 30). Healthy controls within Greater Swiss Mountain Dogs were 56 months (SD, 23) and those with idiopathic epilepsy 46 months (SD, 26) old (overall: 51 months; SD, 25). Eight (35%) of the Border Collies and 12 (52%) of the Greater Swiss Mountain Dogs were female. Although there were no statistically significant differences between dogs with epilepsy and healthy controls regarding baseline characteristics, point estimates indicated a higher proportion of male dogs in the epilepsy group (Table 1). Information regarding the seizure semiology is reported in Table 2 for both breeds.

**Table 1:** Study population.

	Total	No epilepsy	Epilepsy	*p*-value
	*N* = 46	*n* = 15	*n* = 31	
Age [months]	52 (27)	60 (30)	48 (25)	0.15
Female sex	20 (43%)	10 (67%)	10 (32%)	0.055
Weight [kg]	23 (20–48)	39 (21–52)	22 (19–47)	0.23
Breed				
Border Collie	23 (50%)	5 (33%)	18 (58%)	0.21
Greater Swiss Mountain Dog	23 (50%)	10 (67%)	13 (42%)	
Gray matter volume [ml]	55 (52–60)	57 (51–61)	54 (52–60)	0.49
White matter volume [ml]	41 (37–49)	47 (38–59)	38 (36–49)	0.11
Total brain volume [ml]	95 (88–111)	107 (88–115)	92 (88–111)	0.28
Gray matter ratio [%]	58 (56–59)	57 (50–58)	58 (56–59)	0.039*

Data are presented as mean (SD) or median (IQR) for continuous measures, and n (%) for categorical measures. *p*-values indicate statistical significance based on group comparisons between healthy controls and dogs of both breeds with idiopathic epilepsy. Continuous variables were compared using the Mann–Whitney U tests, while categorical and binary variables were analyzed using Fisher’s exact test.

**Table 2:** Seizure semiology.

Medical treatment at the time of MRI		Border Collies [*n*]	Greater Swiss Mountain Dogs [*n*]
Phenobarbital		12	2
Potassium bromide		4	1
Levetirazetam		4	0
Imepitoin		2	1
Type of Therapy	Mono	3	2
	Double	8	1
	Triple	1	0
Drug-naïve		5	10
Time between first seizure and MRI			
< 1 month		3	1
> 1–3 months		3	3
> 3–12 months		5	4
> 12 months		7	5
Time between last seizure and MRI			
> 2 days–1 week		8	3
> 1 week–3 weeks		4	5
> 3 weeks		5	5
N/A		1	0
Seizures			
Status epilepticus		1	0
Cluster seizures		11	0
N/A		2	3
Seizure semiology			
Tonic–clonic		17	6
Focal onset secondary generalization		7	8
Unknown onset		10	3
Additional focal seizures		3	2
Autonomic seizures	Salivation	4	9
	Urination	10	5
	Defecation	7	0
Postictal aggression		2	3
Inter-ictal behavioral changes			
Anxiety		4	3
Over-excited/stressed		1	0

NA, not applicable.

### 3.2 Volumetry

**Figure 2:**
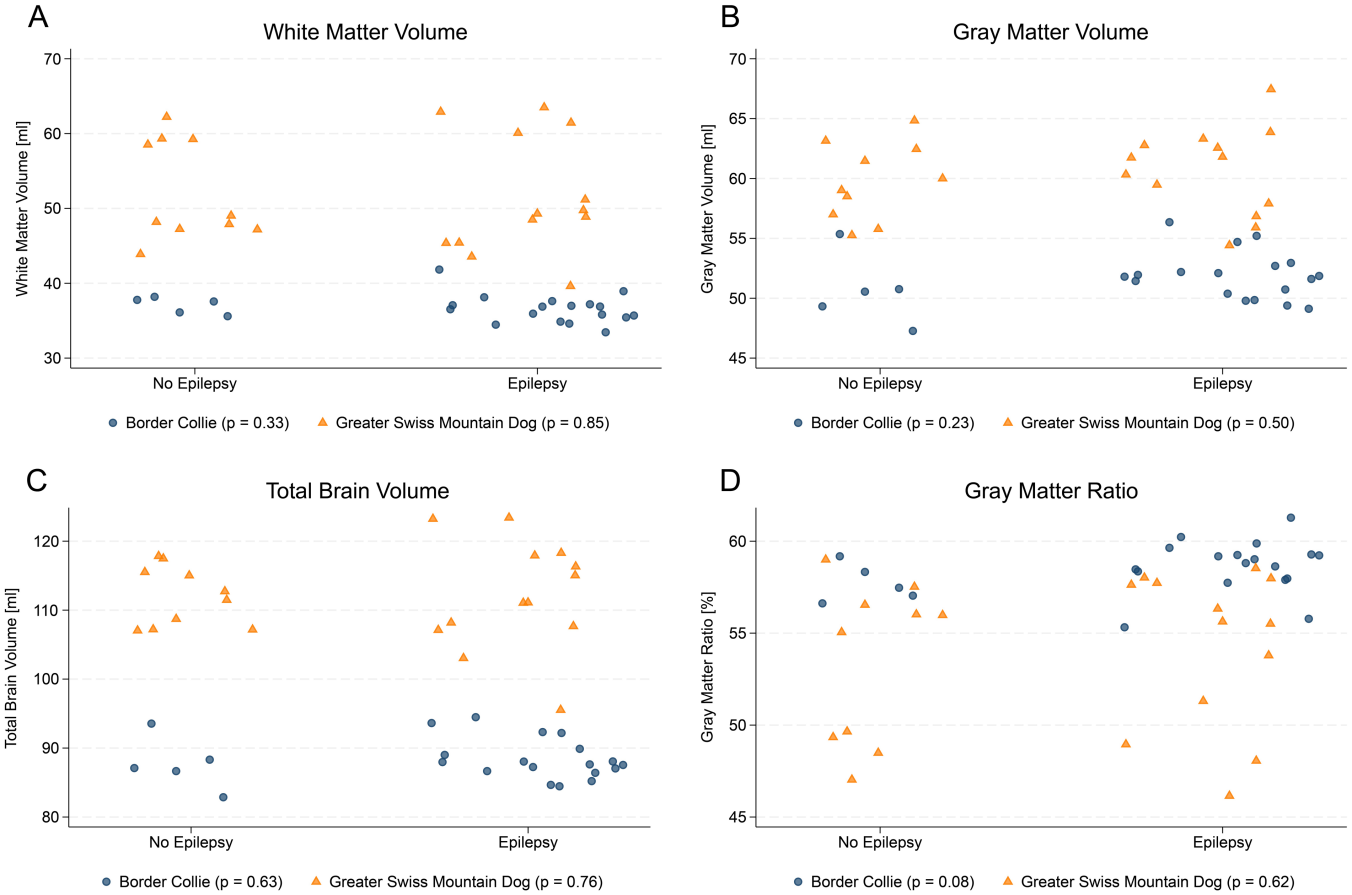
Volumetric analysis of brain structures in dogs with idiopathic epilepsy (*n* = 31) and healthy controls (*n* = 15) stratified by Border Collies (blue circles) and Greater Swiss Mountain Dogs (yellow triangles). Scatterplots illustrate the distribution of **(A)** White Matter Volume (ML), **(B)** Gray Matter Volume (ml), **(C)** Total Brain Volume (ml), and **(D)** Gray Matter Ratio (%). The *p*-values indicate group comparisons between healthy controls and dogs with epilepsy within the respective breed.

Intra-breed volumetric comparisons revealed no significant differences in gray matter volume, white matter volume, total brain volume, or gray matter ratio between healthy controls and dogs with idiopathic epilepsy for either Border Collies or Greater Swiss Mountain Dogs. Between the two breeds, there were distinct baseline differences in brain volumes (Table 1; Figure 2). When pooling data across both breeds, a significantly higher gray matter ratio was detected in healthy controls compared to epileptic dogs (58% vs. 57%, *p* = 0.039). However, this significance was not observed in individual breed analyses (Supplementary Tables 1, 2).

### 3.3 Voxel-based morphometrics

Results of VBM were displayed in statistical parametric maps in FSL Eyes to identify differences in gray matter volume between healthy controls and dogs with idiopathic epilepsy in both breeds.

TFCE analysis, with total brain volume as a covariate followed by FWE-correction (*p* < 0.05) did not reveal any significant differences in gray matter volume between groups. Furthermore, no significant results were obtained through alternative analysis pipelines, including additional analyses with age, sex and disease severity as covariates, TFCE without covariates and FWE-corrected voxel-wise testing.

Consequently, an exploratory analysis was conducted to identify potential neuroanatomical trends. For this purpose, uncorrected t-statistic maps were thresholded at a range of t = 3.1–5.6, corresponding to a voxel-level significance of approximately *p* < 0.001 prior to correction for multiple comparison.

Cluster data were extracted using the FSL Cluster tool, providing voxel volumes, peak t-values and coordinates of the center-of-gravity for each identified region. Anatomical localization was achieved by registering the statistical maps to the Johnson Canine Atlas space using a 12-parameter linear affine registration (FLIRT), allowing for systematic cross-referencing of cluster coordinates with atlas-defined anatomical indices (45).

#### 3.3.1 Border collies

**Figure 3:**
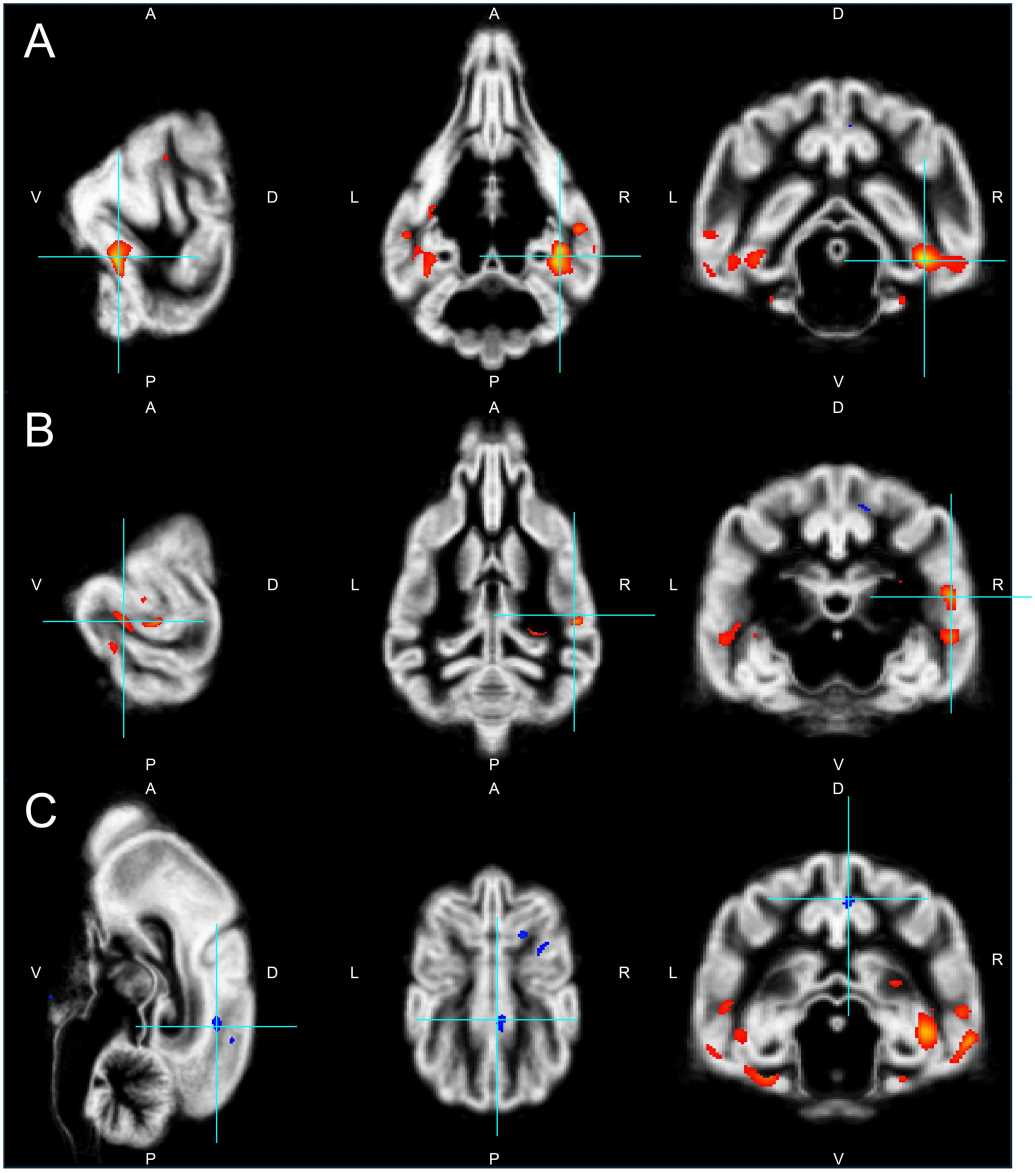
Results of exploratory VBM analysis in Border Collies (idiopathic epilepsy vs. healthy controls). Sagittal, dorsal and transversal views showing key clusters of reduced [B30 **(A)**, B28 **(B)**, colored red] and increased [B42 **(C)**, colored blue] gray matter volume displayed on the study-specific gray matter template. A, anterior; P, posterior; V, ventral; D, dorsal; L, left; R, right.

In the Border Collie cohort, the exploratory analysis revealed distinct patterns of structural alterations (Figure 3; Supplementary Figure 1). Regional gray matter increases were primarily localized to the right retrosplenial fissure (Cluster B30, 2,251 voxels, peak t = 5.52), a key component of the limbic system. Further clusters were identified in the left amygdala (Cluster B25, 156 voxels, peak t = 4.34), the right sylvian gyrus (Cluster B28, 671 voxels, peak t = 4.53) and the left ectosylvian fissure (Cluster B27, 596 voxels, peak t = 4.00). Regional gray matter decreases were found in the right anterior ectolateral gyrus (cluster B44, 446 voxels, peak t = 4.88), the right splenial gyrus (cluster B42, 99 voxels, peak t = 3.45) and in the right motor cortex, specifically in the internal precentral gyrus (cluster B41, 97 voxels, peak t = 3.56).

#### 3.3.2 Greater Swiss Mountain dogs

**Figure 4:**
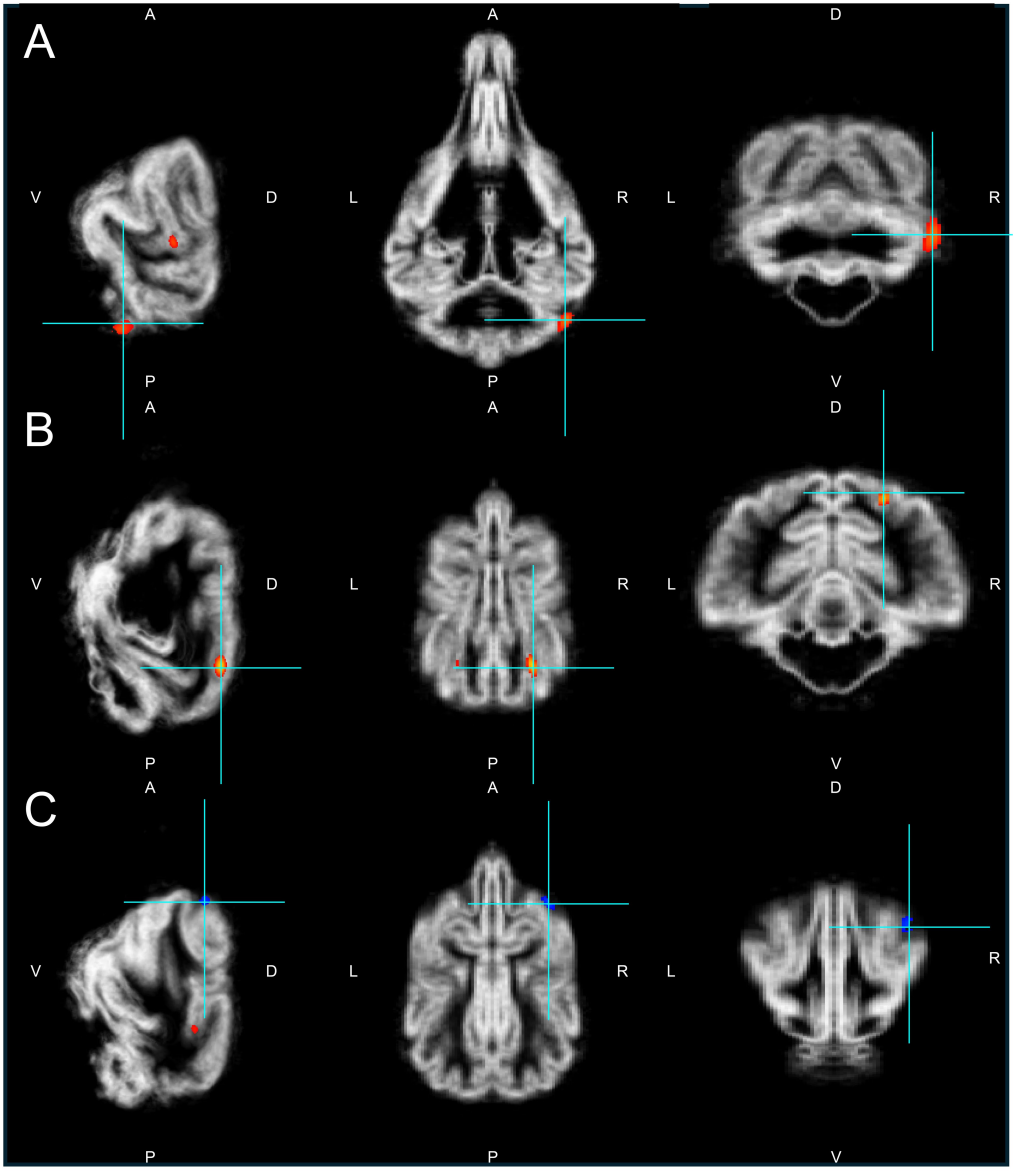
Results of exploratory VBM analysis in Greater Swiss Mountain Dogs (idiopathic epilepsy vs. healthy controls). Sagittal, dorsal and transversal views showing key clusters of reduced [G10 **(A)**, G9 **(B)**, colored red] and increased [G13 **(C)**, colored blue] gray matter volume displayed on the study-specific gray matter template. A, anterior; P, posterior; V, ventral; D, dorsal; L, left; R, right.

Findings in the Greater Swiss Mountain Dog cohort indicated both breed-specific and shared alterations (Figure 4; Supplementary Figure 2). Regarding regional gray matter increases, a prominent cluster was found in the right cerebellum (cluster G10, 720 voxels, peak t = 4.24). Additional findings included clusters in the right sylvian gyrus (cluster G8, 117 voxels, peak t = 3.77) and the right ventral medial recurrent gyrus (cluster G9, 270 voxels, peak t = 5.18). Consistent with the patterns observed in the Border Collie cohort, regional gray matter decreases were detected in the right anterior ectolateral gyrus (cluster G13, 104 voxels, peak t = 3.67) and the left ventral medial recurrent gyrus (cluster G11, 2 voxels, peak t = 3.15).

## 4 Discussion

In this retrospective single-center study, VBM did not reveal significant differences in the gray matter volume between Border Collies and Greater Swiss Mountain Dogs with idiopathic epilepsy and their breed-matched controls. However, exploratory VBM analyses indicated breed-specific clusters of both gray matter volume increases and decreases in Border Collies and Greater Swiss Mountain Dogs, providing a more nuanced view of the possible structural changes associated with idiopathic epilepsy.

VBM has been widely used in neuroradiological research due to its objective, automated approach to detect regional differences in brain tissue volumes across the whole brain. It enables comprehensive, voxel-wise statistical analysis of structural brain changes (13), and has been applied in epilepsy research in both human and veterinary medicine, identifying several regions of altered gray matter volume between healthy individuals and subjects with epilepsy (19, 21, 46, 47).

VBM findings are critically dependent on the underlying image-processing workflow and although objective and automated analysis methods appear to be highly reliable, technical factors influencing such analyses must also be considered. Even in human MRI data, different pipelines and software packages can lead to different results when analyzing the same data (48). In beagle dogs with idiopathic epilepsy, Frank et al. demonstrated reduced gray matter volume in several regions using an SPM-based VBM workflow in contrast to the FSL-based workflow used in the present study (19). It therefore remains unclear whether the difference observed in Beagles is attributable to the workflow or reflects true breed-specific difference; nevertheless, a systematic comparison of the workflows would be desirable for future studies. Methodological reviews emphasize that sample size, segmentation quality, template choice, modulation and smoothing parameters can substantially influence both the pattern and direction of detected effects in VBM (34, 49–51) and therefore, results must be interpreted by taking the underlying pipeline into account. This aspect is especially relevant in dogs, in which variation in skull and brain morphology complicates standardized image processing and therefore the use of VBM (20). To address this limitation, a key strength of the present workflow is the explicit handling of canine brain shape variability through breed- and study-specific templates. In human VBM data-specific templates generally yield higher registration accuracy than general templates (34, 52). Translating this principle to veterinary medicine, restricting the analysis to a distinct dog breed may help improve the robustness of VBM in epilepsy (19). By creating separate templates for Border Collies and Greater Swiss Mountain Dogs, this study further reduced registration errors arising from interbreed anatomical differences. Not surprisingly, the comparison of brain volume ratios revealed differences between the two breeds studied. Previous studies have already demonstrated the significant influence of breed on brain volume, and our findings, particularly regarding differences in the gray matter volume ratio, support breed-specific analyses (17, 53).

To obtain a preliminary overview, in addition to VBM, we examined the total volumes of gray and white matter, total brain volume, and the corresponding ratios. Intra-breed volumetric comparisons yielded no significant differences between healthy and epileptic dogs. Notably, a statistically significant difference in gray matter ratio emerged exclusively when pooling data across both breeds. However, this finding is likely the consequence of a confounding statistical artifact driven by baseline differences between the two breeds and an uneven group composition, rather than a genuine feature of idiopathic epilepsy. Specifically, of the 31 dogs diagnosed with idiopathic epilepsy, 58% were Border Collies, and 42% were Greater Swiss Mountain Dogs. In contrast, of the 15 healthy controls 67% were Greater Swiss Mountain Dogs and 33% Border Collies. This methodological pitfall underscores the critical importance of breed-specific evaluations in veterinary morphometric research.

Our semi-manual brain extraction with visual quality control provides an additional safeguard and is consistent with recommendations stating that accurate masking and atlas-based segmentation are essential for reliable canine morphometry (20). Although our semi-manual brain extraction was carefully quality-controlled, fully automated and standardized pipelines would increase objectivity and reproducibility.

Spatial smoothing represents another step within the VBM preprocessing workflow, though no standardized Gaussian kernel size has been established for dogs. Human VBM often uses 6–12 mm kernels (48, 51, 54), while previous studies in dogs have used a 2 mm kernel to compensate for the smaller brain size and finer anatomical structures compared with humans (19, 24). This smaller Gaussian kernel is supported by non-human primate work suggesting that a smaller kernel size is appropriate in non-human brains (55, 56). Given the voxel size of 0.5 mm, we selected a 1.5 mm Gaussian kernel to achieve a favorable balance between spatial resolution and noise reduction, thereby enabling the detection of small clusters while minimizing artifacts.

In our study, conservative whole-brain FWE-correction did not reveal statistically significant gray matter volume differences between epileptic dogs and healthy controls. However, exploratory analysis using a t-score threshold range of 3.1–5.6 identified biologically plausible neuroanatomical trends, with clusters of increased gray matter volumes within the limbic and temporal regions and focal gray matter reductions in neocortical and motor areas. These subtle changes may be consistent with localized reductions in fractional anisotropy (28) indicating subtle microstructural white matter impairment in a similar cohort of dogs using diffusion tensor-based analysis. Similar to the present study, the previous voxel-based diffusion tensor imaging analysis also failed to yield significant results after whole-brain correction. Significant results were obtained only when specific regions were selected. The coexistence of white matter microstructural changes and gray matter volume alterations suggests that idiopathic epilepsy in these breeds may manifest as network-restricted structural remodeling rather than diffuse pan-cortical degeneration.

Traditionally, epilepsy is described with neurodegeneration and subsequent gray matter atrophy. VBM and meta-analysis studies in idiopathic generalized epilepsy, juvenile myoclonic epilepsy and temporal lobe epilepsy have reported gray matter volume reductions in the thalamus, hippocampus, insula caudate, cerebellum, occipital and frontal cortex and the putamen in humans (16, 46, 57–62). Veterinary VBM studies describe similar patterns in dogs, including decreased gray matter volume in the olfactory bulb, parietal and temporal cortices, hippocampus and cingulate gyrus, consistent with epilepsy-associated cortical and limbic atrophy in dogs (19, 63, 64). In line with these findings, our exploratory analysis identified focal volume reduction in neocortical and motor areas, as well as a shared finding of gray matter reduction in the limbic-prefrontal network (right anterior ectolateral gyrus) in Border Collies and Greater Swiss Mountain Dogs.

At the same time, we observed breed-specific volume increases, with Border Collies showing predominant limbic and temporal involvement, and Greater Swiss Mountain Dogs exhibiting cerebellar increases. This is consistent with human VBM studies on epilepsy, which demonstrate areas of increased gray matter volume alongside areas of focal volume loss, which are increasingly understood as markers of large-scale network reorganization rather than indicators of purely focal atrophy (16, 57–60). Reported anatomical regions with increased gray matter volume in patients with epilepsy include the medial and superior frontal and anterior cingulate, precentral gyrus, amygdala, parahippocampal/fusiform and precuneus/cuneus regions (16, 47, 57–60, 65). Furthermore, in the only VBM study in dogs with idiopathic epilepsy to date, Frank et al. found both reduced and increased gray matter volume in the frontal/parietal cortex and piriform lobe (19). The structural abnormalities identified here in cingulate and frontal regions correspond to the increased functional connectivity within the anterior default mode network that has been previously demonstrated in the same cohort. This regional volume increase may be considered a macroscopic structural correlate of increased connectivity (30). Our exploratory clusters, particularly in limbic, frontal and cerebellar regions, are therefore broadly consistent with a distributed pattern of both volume increase and decrease within epileptic networks.

Notwithstanding the methodological strengths of this tailored workflow, several limitations must be acknowledged. The primary constraint is the limited sample size. While a breed-specific VBM analysis likely reduced anatomical noise, it also limited statistical power and may have contributed to the absence of FWE-corrected group differences (13, 14). Despite the absence of significant differences in demographic characteristics between epileptic dogs and healthy controls, point estimates indicated a higher proportion of male dogs in the epilepsy group, which could potentially have confounded the morphometric results. Consequently, subsequent studies should prioritize sex-matched cohorts to minimize demographic variability.

Furthermore, the reliance on first-degree relatives as controls may have introduced bias due to shared genetic predisposition. The mean age of the control group at inclusion was 60 months (5 years; Table 1), which exceeds the reported mean age of onset for idiopathic epilepsy, typically ranging between 2 and 4.5 years (6, 66, 67). It also exceeds the specific onset ages for Border Collies (2.8 years) (68), and Greater Swiss Mountain Dogs (2.4 years) (4). As age-related changes in gray matter volume have been identified in dogs (24), we cannot completely rule out the possibility that the relatively advanced age of the healthy control dogs influenced the analysis, even though additional analyses with age and sex as covariates did not confirm this effect. Interestingly, the dogs in this study had the opposite age distribution compared with that in the previous VBM study of dogs with idiopathic epilepsy by Frank et al., in which control dogs were, on average, only half as old as dogs with idiopathic epilepsy. This difference may also have contributed to the divergent results between the two studies.

Lastly, arguably the most critical limitation of our study is that the dogs were scanned at a single timepoint, without longitudinal follow-up to monitor potential changes over time. As a large proportion of the dogs were examined shortly after seizure onset, we cannot rule out the possibility that further changes may have emerged as the condition progressed, possibly depending on disease severity.

Future work should prioritize larger, possibly multi-center cohorts to increase statistical power and enable robust FWE-corrected analyses, ideally integrating structural, diffusion and functional imaging. However, this is also one of the main challenges in veterinary neuroimaging. Ideally, a large, publicly accessible canine imaging database would be needed, including both high-quality imaging data and relevant clinical information in a standardized format. Such a resource could facilitate the future application of artificial intelligence and machine-learning approaches to automated workflows, such as those used for brain extraction (69), as well as to more complex analyses. Such studies will be essential to validate whether the neuroanatomical trends identified in our study constitute reliable markers of idiopathic epilepsy across different canine populations and to clarify the relative contributions of white matter loss, inflammatory processes and network reorganization to gray matter volume alterations in epilepsy.

## 5 Conclusion

In this retrospective single-center MRI study, FWE-corrected VBM did not reveal significant volume alterations in gray matter between dogs with idiopathic epilepsy and breed-matched healthy controls. Mild findings in exploratory VBM analyses indicate a mixed pattern of focal gray matter decreases in neocortical and motor regions alongside localized gray matter increases in limbic, frontal, temporal and cerebellar areas. Volumetric analyses did not differ significantly between healthy controls and dogs with idiopathic epilepsy within each breed but revealed distinct baseline differences between Border Collies and Greater Swiss Mountain Dogs. Future large-scale studies are warranted to externally validate these findings and assess their relevance to canine epilepsy.

## Data Availability

The datasets used and analyzed during the current study are available from the corresponding author on reasonable request.

## Footnotes

1
www.nitrc.org/projects/mricron

2
www.slicer.org

3
www.itksnap.org
