# Supplementary material for: Investigating gray matter volumes in idiopathic epileptic Border Collies and Greater Swiss Mountain Dogs using voxel-based morphometry
Source: Front Vet Sci. 2026 Jul 17;13:1872171. doi: 10.3389/fvets.2026.1872171 (PMC13470667; doi:10.3389/fvets.2026.1872171)
Supplement: Supplementary file 1 [file Data_Sheet_1.pdf]

Supplementary Material

1 Supplementary Analyses

As requested by the reviewer, a sensitivity analysis was performed to validate our primary findings. The VBM pipeline was re-run separately for both breeds, using an alternative approach where a single study subject served as the study-specific template. The template subject was selected from the healthy control cohort in accordance with the criteria detailed in Section 2.4.2 to ensure an optimal signal-to-noise ratio. Consistent with the main analysis, this additional validation revealed no statistically significant results, confirming that the choice of template construction did not alter the core outcomes of the study.

2 Supplementary Figures

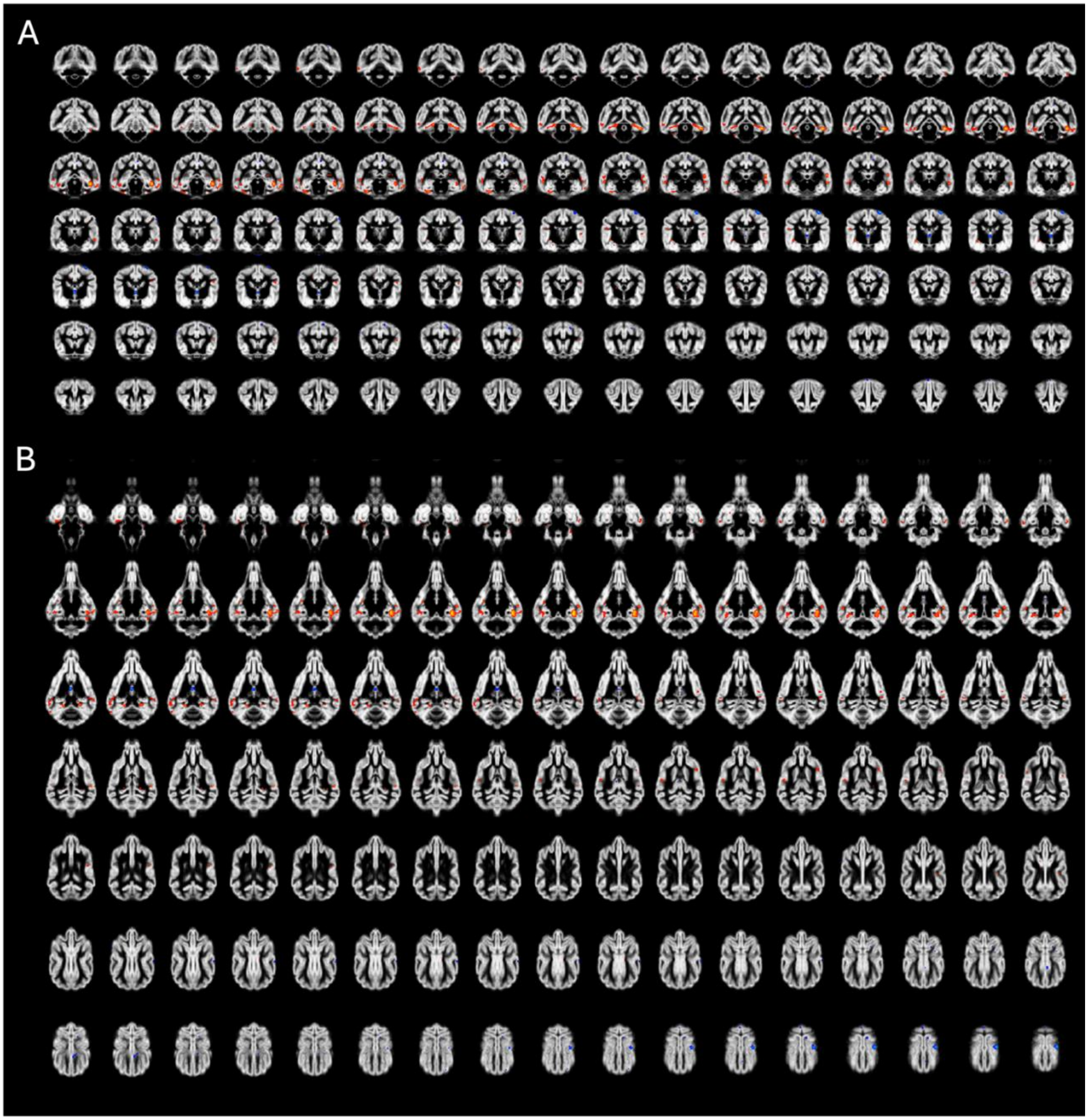

Supplementary Figure 1. Results of exploratory VBM analysis in Border Collies (idiopathic epilepsy vs. healthy controls). Transversal (A) and dorsal (B) views showing regions of reduced (colored red) and increased (colored blue) gray matter volume in the epileptic dogs compared to healthy individuals displayed on the study-specific gray matter template.

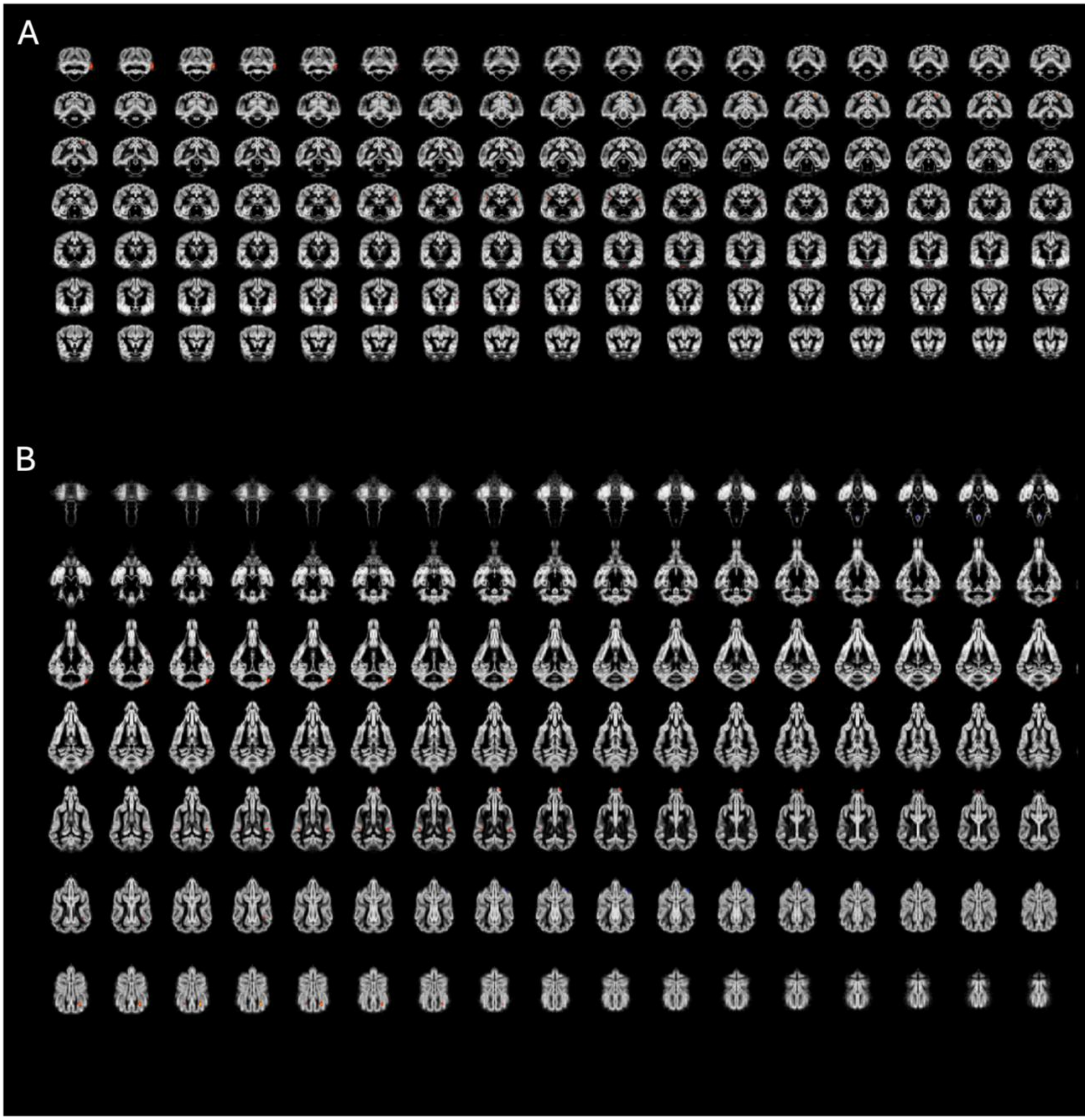

Supplementary Figure 2. Results of exploratory VBM analysis in Greater Swiss Mountain Dogs (idiopathic epilepsy vs. healthy controls). Transversal (A) and dorsal (B) views showing regions of reduced (colored red) and increased (colored blue) gray matter volume in the epileptic dogs compared to healthy individuals displayed on the study-specific gray matter template.

3 Supplementary Tables

Supplementary Table 1. Study Population, Border Collie Group

	**Total**	**No Epilepsy**	**Epilepsy**	**p-value**
	**N=23**	**N=5**	**N=18**	
Age [months]	54 (30)	70 (43)	49 (25)	0.18
Female sex	8 (35%)	3 (60%)	5 (28%)	0.30
Weight [kg]	20 (16-21)	20 (20-21)	20 (16-21)	0.60
Gray matter volume [ml]	52 (50-53)	51 (49-51)	52 (50-53)	0.23
White matter volume [ml]	37 (36-38)	38 (36-38)	37 (35-37)	0.33
Total brain volume [ml]	88 (87-90)	87 (87-88)	88 (87-90)	0.63
Gray matter ratio [%]	59 (58-59)	57 (57-58)	59 (58-59)	0.080

Data are presented as mean (SD) or median (IQR) for continuous measures, and n (%) for categorical measures.

Supplementary Table 2. Study Population, Greater Swiss Mountain Dog Group

	**Total**	**No Epilepsy**	**Epilepsy**	**p-value**
	**N=23**	**N=10**	**N=13**	
Age [months]	51 (25)	56 (23)	46 (26)	0.38
Female sex	12 (52%)	7 (70%)	5 (38%)	0.21
Weight [kg]	49 (42-55)	48 (39-54)	50 (42-58)	0.25
Gray matter volume [ml]	60 (57-63)	60 (57-62)	62 (58-63)	0.50
White matter volume [ml]	49 (47-59)	49 (47-59)	49 (45-60)	0.85
Total brain volume [ml]	111 (107-117)	112 (107-116)	111 (108-118)	0.76
Gray matter ratio [%]	56 (49-58)	56 (49-57)	56 (51-58)	0.62

Data are presented as mean (SD) or median (IQR) for continuous measures, and n (%) for categorical measures.
